# Multi-level policies for air quality: implications of national and sub-national emission reductions on population exposure

**DOI:** 10.1007/s11869-018-0613-1

**Published:** 2018-09-06

**Authors:** Emanuela Peduzzi, Enrico Pisoni, Alain Clappier, Philippe Thunis

**Affiliations:** 1 European Commission, Joint Research Centre (JRC), Ispra, Italy; 2 Laboratoire Image Ville Environnement, Université de Strasbourg, 3, rue de l’Argonne, 67000 Strasbourg, France

**Keywords:** Air quality (AQ), Integrated assessment model (IAM), PM_2.5_ exposure, Source receptor relationships (SRR), Surrogate modelling, Metamodelling

## Abstract

Poor air quality and related health impacts are still an issue in many cities and regions worldwide. Integrated assessment models (IAMs) can support the design of measures to reduce the emissions of precursors affecting air pollution. In this study, we apply the SHERPA (screening for high emission reduction potentials for air quality) model to compare spatial and sectoral emission reductions, given country-scale emission targets. Different approaches are tested: (a) country ”uniform” emission reductions, (b) emission reductions targeting urban areas, (c) emission reductions targeting preferential sectors. As a case study, we apply the approaches to the implementation of the National Emission Ceiling Directive. Results are evaluated in terms of the reduction in average population exposure to PM_2.5_ overall in a country and in its main cities. Results indicate that the reduction of population exposure to PM_2.5_ highly depends on the way emission reductions are implemented. This work also shows the usefulness of the SHERPA model to support national authorities implementing national emission reduction targets while, at the same time, addressing their local air quality issues.

**Electronic supplementary material:**

The online version of this article (10.1007/s11869-018-0613-1) contains supplementary material, which is available to authorized users.

## Introduction

Integrated assessment models (IAMs) are increasingly used to support the development of air quality (AQ) policies (Thunis et al. 2016b). They provide a simplification of reality that allows users to simulate and connect complex phenomena. They can therefore be used to evaluate, for example, policy scenarios and their consequent emission reductions in terms of pollutant concentrations changes and environmental, health and economic impacts. IAMs applied to AQ can provide (i) an analysis of exceedances (reasons for air quality non-compliance), (ii) details of possible measures and the time required to attain the reduction objectives, (iii) cost-benefit/cost-effectiveness analysis, (iv) information on the impact on human health and the environment and (v) information on related uncertainties and robustness (Viaene et al. 2016).

Important components of IAMs for AQ are the *emission inventory* and the *surrogate model*. The emission inventory provides the distribution of emissions (by precursor, by cell, country or region) to which reductions can be applied (as a consequence of a policy scenario or of emission reduction measures). The surrogate model is normally a simplification of complex chemical transport models (CTMs) that allows the user to rapidly evaluate the pollutant concentration changes (by cell, sector, region or country). Several surrogate modelling approaches applied to AQ are reviewed by Clappier et al. (2015). Surrogate models are developed on the basis of a set of full CTM simulations. The number and type of simulations performed within this set and the type of surrogate model will determine the range of application and the flexibility of the surrogate model to address specific issues. The challenge is to cover the widest range of applicability for the lowest number of full CTM simulations (which are time-consuming).

IAMs used for policy support generally rely on surrogate models represented by so-called *source-receptor relationships* (SRRs). These SRRs consist of linear relationships between emission reductions applied at country/regional level and the concentration changes in each cell of the grid (country/precursor-to-grid) or the corresponding country averages concentration changes (country/precursor-to-country).

Examples of these IAMs are the GAINS (Greenhouse Gas–Air Pollution Interactions and Synergies) model (Amann et al. 2011) and the FAst Scenario Screening Tool (TM5-FASST) model (Van Dingenen et al. 2018). GAINS relies on a reduced form representation of the European Monitoring and Evaluation Programme/Meteorological Synthesizing Centre-West (EMEP/MSC-W) Eulerian model (on a ∼ 28 × 28 km^2^ grid) (Simpson et al. 2012) combined with a downscaling methodology for the assessment of PM (particulate matter) and NO_2_ concentrations at urban level (Kiesewetter and Amann 2014). It is also available for country-specific analysis, as for example GAINS-Italy, taking into account 20 subnational regions (Ciucci et al. 2016). FASST mimics the full TM5-CTM global model based on 56 source-receptor regions (Van Dingenen et al. 2018). In particular, the GAINS model, developed at the International Institute for Applied Systems Analysis (IIASA), was used for the revision of the National Emission Ceiling Directive (DIRECTIVE 2001/81/EC 2001; Amann 2014). The revision led to the replacement of the directive with the *new* National Emission Ceilings (NEC) Directive (DIRECTIVE (EU) 2016/2284 2016), which aims to achieve the objectives of the European Commission’s Thematic Strategy on Air Pollution (TSAP).

IAMs can also be used at the city and regional scales (Relvas and Miranda 2018) to support the implementation of the Ambient Air Quality Directive (AQD) (DIRECTIVE 2008/50/EC 2008). This directive aims at “defining and establishing the objectives for ambient air quality designed to avoid, prevent or reduce the harmful effects on human health and the environment” (DIRECTIVE 2008/50/EC 2008). In order to achieve these objectives, member states (MS) are required to assess and monitor AQ and in case of non-compliance to design appropriate air quality plans (AQPs) and to cooperate with other member states to reduce air pollution. These AQPs should outline the abatement measures to be implemented and the expected improvement in AQ (Viaene et al. 2016; Carnevale et al. 2012).

Member states need to comply, at national level, to the NEC Directive (DIRECTIVE (EU) 2016/2284 2016) and at the same time fulfil the AQ objectives/standards set by the AQD at the regional and city scales (Guariso et al. 2016). In this context, it is therefore important to consider the spatial variability of emissions in an integrated way (Ferreira et al. 2017).

For this purpose, the Joint Research Centre (JRC) recently developed SHERPA (screening for high emission reduction potentials for air quality) (Thunis et al. 2016a), a tool which relies on a novel spatially specific (or flexible) cell-to-cell SRRs described in Pisoni et al. (2017). These SRRs require a limited number of training runs (between 15 and 20) and allow users to differentiate the effects of emission reduction scenarios by sector and for any given area. SHERPA can be used to estimate the concentration response of particulate matter (PM_2.5_, PM_10_) and NO_2_ due to a decrease in emissions of the precursors: primary particulate matter (PPM), NO_x_, volatile organic compounds (VOC), SO_2_ and NH_3_. The SRRs are designed for validity within the current legislation (CLE) and the maximum technically feasible emission reduction (MTFR) scenarios as described in Amann et al. (2014). The average yearly concentration changes are evaluated on a ∼ 7 × 7 km^2^ grid covering Europe. The emission reductions can be defined by macro-sector (such as energy, residential, traffic, agriculture etc.) and/or control areas defined by the European Nomenclature of Territorial Units for Statistics (NUTS) covering levels ranging from the country scale (NUTS0) to the local scale (NUTS2 and NUTS3). Control areas can also be functional urban areas (FUAs) as defined by the EU-OECD (EU-OECD 2013). FUAs include *city cores* and *commuting zones* and identify the urban hinterlands whose labour market is highly interconnected with the city cores, normally going beyond the limits of the urban administrative units.

SHERPA is therefore designed to support local and regional authorities in addressing their AQ issues and designing their AQPs. The spatial detail of the SRRs allows (i) the evaluation of emission reduction scenarios by sector and area, (ii) the source allocation of pollution originating from inside a specific area in terms of sectors and precursors and of pollution originating elsewhere and (iii) the identification of the principal source areas of pollution at a location (governance).

The purpose of this work is to assess how the assumptions behind linear SRRs, used in IAMs, can impact the outcome of air quality policies, at both the national and local scales. In particular:

- The first objective is to analyse how the added flexibility of the SRRs described in Pisoni et al. (2017) can impact air quality policies. The analysis is carried out by using the SRR model to evaluate the impact of different emission reduction scenarios on average PM_2.5_ concentration and exposure. The impacts of emission reductions in terms of sectors or urban/non-urban areas are compared to uniform percentage emission reductions over a country (i.e., for each precursor reduction, all sectors are treated together), equivalent to a country-to-grid SRR approach.
- The second objective is to analyse, as a case study, the application of the NEC Directive (DIRECTIVE (EU) 2016/2284 2016) within a country using the spatially specific SRR model described in Pisoni et al. (2017). This directive sets emission reduction commitments per precursor and per member state for the years 2020 and 2030 with respect to the year 2005. It is therefore interesting to evaluate the variability of the impacts, both at country level and at city level, that can be obtained by applying the reductions in precursor emissions across areas and sectors.

## Methodology: impact of SRR spatial detail on AQ policies

The analysis of the spatially specific SRR model is carried out by evaluating the impacts of emission reductions in specific areas and specific sectors. Emission reductions are applied only to the grid cells belonging to a given sector or given country subarea (hereafter the sector/area combination affected is referred to as *source*), and evaluating the average impacts on concentration and exposure on the whole country or in a specific area (hereafter the area where the impacts are evaluated is referred to as *receptor* area). The impacts, which are either normalised or equivalent in terms of total emission reductions, are compared to those obtained by applying uniform emission reductions (for a given precursor) to *all grid cells* belonging to a country (source) as in a country-to-grid SRR approach. Results are finally compared using relevant indicators.

**Fig. 1:**
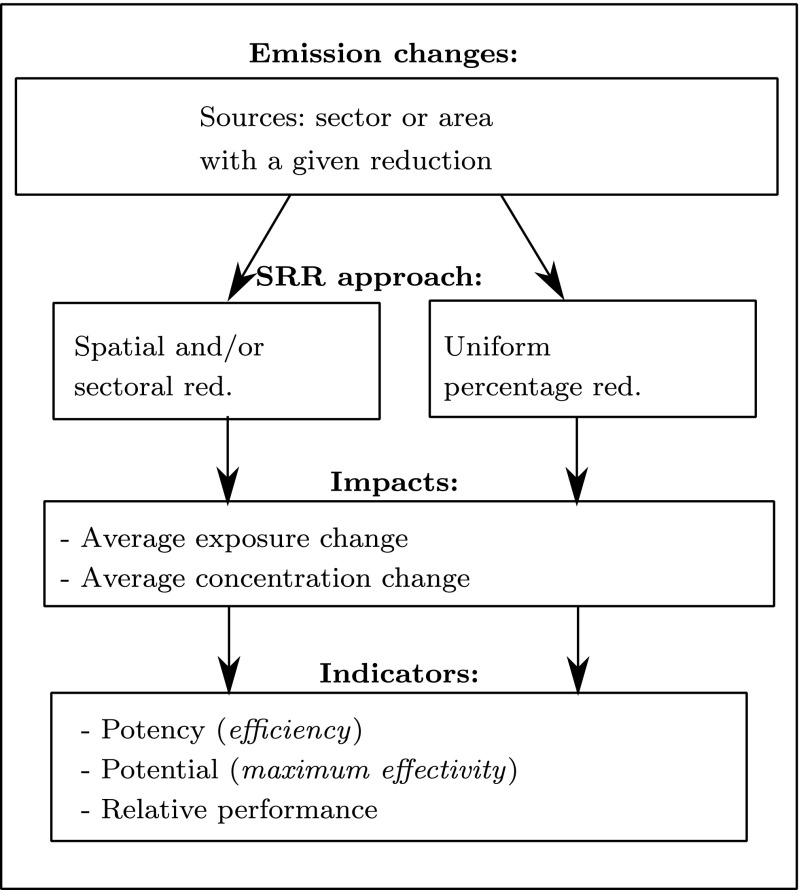
Overview of the methodology used to evaluate the spatially specific SRRs

The methodology and the steps carried out in this analysis are summarised in Fig. 1 and explained in more detail in the following paragraphs.

### Sources (sectoral and spatial) and receptors

As said earlier, the term *source*, *s*, refers to the sectors whose emissions are reduced (e.g. transport) and the area where the reductions occur (i.e the urban areas or the non-urban areas within a country, or the whole country).

In order to systematically evaluate spatial reductions, the urban and non-urban areas of each country are distinguished. Urban areas are identified according to the EU-OECD definition of FUA. For each country, all the FUAs defined by OECD (2013) are considered. Each country can therefore be divided into an area belonging to FUAs and *the rest of the country*, outside any FUA area (pseudo-rural areas).

In terms of emission inventory, 10 activity macro-sectors corresponding to the Selected Nomenclature for Air Pollutants (SNAP) CORINAIR standard are considered. Total national emissions for each of these sectors and for the year 2010 are those given by IIASA in the framework of the Thematic Strategy on Air Pollution (scenario: PRIMES 2013 REF-CLE (ID: TSAP_Sept2013_P13_REFv3), Energy Systems Model of the National Technical University of Athens, in its reference scenario under the current legislation). These emissions are gridded to the same resolution as the grid of the underlying SRR (∼7 × 7 km^2^) using proxies derived from the MACC-TNO emission inventory (Kuenen et al. 2014), population density and the EC4MACS project (EC4MACS 2013). For convenience, the 10 macro-sectors are aggregated into five sectors (industry, residential, transport, agriculture, other) as shown in Table 1.

**Table 1:** Definition of the aggregated sectors corresponding to the SNAP CORINAIR standards

Aggregated sectors	Corresponding SNAPs
Industry	1, 3, 4
Residential	2
Transport	7
Agriculture	10
Other	5, 6, 8, 9

The term *receptor*, *r*, refers to the area where the impacts are evaluated, which, in this study, can be a whole country or the grid cell corresponding to the centroid of a FUA.

### Emission changes

Impacts on air quality are evaluated, in a given country, as a consequence of a variation in the emissions, Δ*E*_*p*,*s*_, of a precursor, *p* (PPM, NO_x_, SO_2_, NH_3_, NMVOC), of a given *source*, *s*. The absolute value of emission change is expressed in Eq. 1.

1 $$ {\Delta} E_{p,s} = R_{p,s} \cdot \sum\limits_{i} E_{i,p,s} \cdot A_{i} \cdot F_{i,s} = \sum\limits_{i} {\Delta} E_{i,p} \cdot A_{i} \cdot F_{i,s} $$

where *E*_*i*,*p*,*s*_ is the emission density for each precursor *p* (emissions in kton/km^2^) relative to the source, *s*, in each grid cell, *i*, belonging to the area where the reduction of emission is applied. *R*_*p*,*s*_ is the reduction fraction that is applied to *E*_*i*,*p*,*s*_. *A*_*i*_ is the area of each grid cell, and *F*_*i*,*s*_ is the cell fraction belonging to the *source* area, where the emission reduction is applied. As said earlier, with the spatially specific SRR approach, reductions are applied *only to the cells* corresponding to the sources of the emissions (the sectors, the FUA areas or non-FUA areas), whereas with uniform reductions, the same percentage emission reduction is applied *to all cells* within a country independently of the area or sector that is the source.

### SRR calculation procedure

Given the emission change, Δ*E*_*i*,*p*_ in each source cell, *i*, for each precursor, *p*, the spatially specific SRR proposed by Pisoni et al. (2017) is applied to calculate the corresponding concentration change, Δ*C*_*j*,*p*_, in each receptor cell *j* of the target area *r*. The SRR model is represented by Eq. 2.

2 $$ {\Delta} C_{j,p} = {\sum\limits_{i}^{N}} \alpha_{j,p} \cdot (1+d_{i,j})^{-\omega_{j,p}} \cdot {\Delta} E_{i,p} $$

where *d*_*i*,*j*_ is the distance between the source cell *i* and the receptor cell *j*. *α*_*j*,*p*_ and *ω*_*j*,*p*_ are the amplitude and width of the “bell shape” function, and they describe, respectively, the relative importance of each precursor *p* to the pollutant concentration in cell *j* and its decrease with distance (see details in Pisoni et al. 2017). These two parameters, for each cell and for each precursor, are obtained by *training* this model with the annual mean pollutant concentrations obtained from the CHIMERE air quality model (Menut et al. 2013), with the procedure also described in Pisoni et al. (2017). In the present work, the pollutant concentration considered is yearly averaged PM_2.5_. The SRR model is linear and therefore the effect on the concentration in cell *j* for all the precursors considered, *P*, is the sum of the contribution of each precursor as shown in Eq. 3.

3 $$ {\Delta} C_{j} = {\sum\limits_{p}^{P}} {\Delta} C_{j,p} $$

As the SRR works on emission reduction scenarios, the only way to validate it is through comparison of the concentration levels obtained from a series of emission reduction scenarios with the results obtained from the same scenarios from the full CTM model (CHIMERE) (independent from those used to train the model) . The validation of the SRR model used in this study is reported in Pisoni et al. (2017) and in Thunis et al. (2018). Results in Pisoni et al. (2017) show that the SRR is able to reproduce the chemical processes of the CTM and that its relative bias (compared to the CTM) is typically less than 5% in most validation areas though it may reach 10% at some locations, mostly mountains. The robustness of the SRR model used in this study is evaluated in Pisoni et al. (2018).

### Impacts

The impacts are represented by the average PM_2.5_ exposure change, Δ*I*_*p*,*r*_ (population-weighted concentration change), in a given *receptor* area (*r*). Δ*I*_*p*,*r*_ is calculated from the concentration change in each grid cell (Δ*C*_*j*,*p*_) as shown in Eq. 4.

4 $$ {\Delta} I_{p,r} = \frac{{\sum}_{j} {\Delta} C_{j,p} \cdot P_{j} \cdot F_{j,r}}{{\sum}_{j} P_{j} \cdot F_{j,r}} $$

where *P*_*j*_ is the population in each cell and *F*_*j*,*r*_ is the fraction of each cell belonging to the receptor area (*r*). The population data from the LUISA platform (Aurambout and Lavalle 2015) is adapted to the ∼7 × 7 km^2^ grid.

The indicators which will be described in “Indicators” and all results presented in “Results: impacts of SRR assumptions on air quality policies” refer to Δ*I*_*p*,*r*_.

The exposure change is the indicator preferred in this study to describe average impacts as it is related to the health outcome of reduction scenarios (World Health Organization Europe 2013). The analysis however can be carried out also considering the average PM_2.5_ surface-weighted concentration change, Δ*C*_*p*,*r*_, as defined in Eq. 5 for a given precursor *p* and in a given *receptor* area , where *A*_*j*_ represents the area of each cell. Corresponding results are reported in the Appendix.

5 $$ {\Delta} C_{p,r} = \frac{{\sum}_{j} {\Delta} C_{j,p} \cdot A_{j} \cdot F_{j,r}}{{\sum}_{j} A_{j} \cdot F_{j,r}} $$

The impact of a specific reduction scenario on a single cell is evaluated considering the concentration change in that cell due to all precursors, as shown in Eq. 3.

### Indicators

Different indicators are used in this study to evaluate the *effectiveness* of emission reductions from different sources (sectors and areas) and precursors relatively to equivalent reductions applied to the whole country. These indicators are the *potency*, the *relative potential* and the *relative performance ratio*. Because of the linearity of the SRR model, these indicators, when defined per precursor and per source, do not depend on the reduction fraction *R*_*p*,*s*_ that is applied to the emissions (1).

The *potency* (or *efficiency*), *η*_*p*,*s*_, as introduced by Clappier et al. (2017), of each source (*s*) and precursor (*p*) is expressed in Eq. 6. *η*_*p*,*s*_ is a finite derivative that provides information on the speed of change in terms of impact, per unit change of emissions for a given source.

6 $$ \eta_{p,s} = \frac{{\Delta} I_{p,r}}{{\Delta} E_{p,s}} $$

In the case of uniform emission reductions, the potency only depends on the precursor being reduced (for each country) while it does not depend on the sector or country sub-region (urban and non-urban areas), i.e., where the reduction is applied. The potency, in this case, is expressed by Eq. 7.

7 $$ \eta_{p,country} = \frac{{\Delta} I_{p,r}}{{\Delta} E_{p,country}} $$

where Δ*E*_*p*,*country*_ is obtained, as said earlier, by applying the same reduction fraction *R*_*p*,*s*_ to all cells belonging to the country in question.

The *relative potential* (or *maximum effectivity*) as defined by Clappier et al. (2017) , *ϕ*_*p*,*s*_, is expressed in Eq. 8. It provides information on the maximum (*relative*) impact reached when all emissions (within the range of validity of the SRR model) are *switched off* for a given source area or precursor-sector combination. In Eq. 8, *I*_*r*_ is the exposure due to the PM_2.5_ concentration in each cell of the receptor area, *r*, in the baseline scenario (which refers to the year 2010).

8 $$ \phi_{p,s}= \frac{{\Delta} I_{p,r}}{I_{r}} \cdot \frac{E_{p,s}}{{\Delta} E_{p,s}}=\frac{\eta_{p,s} \cdot E_{p,s}}{I_{r}} $$

In the case of uniform emission reductions, for each precursor, the contribution of each source to the impacts can be expressed as shown in Eq. 9.

9 $$ \phi_{p, s, country}= \frac{\eta_{p,country} \cdot E_{p,s}}{I_{r}} $$

The indicators defined in Eqs. 6 and 7 depend only on the SRR approach used (as they are normalised on the reduction of emissions), whereas those defined in Eqs. 8 and 9 depend on both the SRR model and the underlying emission inventory. It is important to note that both indicators are useful for the interpretation of the results. While the potency highlights the speed of change, the relative potential refers to the maximum (*relative*) achievable impact (within the range of validity of the model). Low potencies (e.g. due to non-efficient chemical transformations) could correspond to high potentials where the amount of emissions is important, and vice versa.

It should be kept in mind that *potency* and *relative potential* estimated using a linear SRR are validated only for emission reductions within the range of validity of the model for which the linearity assumption is verified. In particular, the relative potential should be interpreted, as said earlier, as the relative potential of emission reductions within this range. Extending these values to the whole range of emissions can lead to a biased estimate. A telling example is that of NH_3_ emissions from agriculture being a limiting factor for secondary PM formation when interacting with high NO_x_ emissions in urban areas. This issue is well highlighted in Thunis et al. (2018) with a very simple example, which evaluates the attribution of secondary PM formation to NH_3_ and NO_x_ for different precursor reduction scenarios under linear and non-linear conditions.

The *relative performance ratio*, *ρ*_*p*,*s*_, defined in Eq. 10, represents the ratio between the impact obtained with the spatially specific SRR approach and that obtained from equivalent uniform emission reductions across the country. For each source and precursor, the relative performance ratio is equivalent to the ratio between *η*_*p*,*s*_ and *η*_*p*,*country*_ (6 and 7) and the ratio between *ϕ*_*p*,*s*_ and *ϕ*_*p*,*s*,*country*_ (8 and 9).

10 $$ \rho_{p,s} = \frac{{\Delta} I_{p,s}}{{\Delta} I_{p,country}}|{~}_{{\Delta} E_{p,s}={\Delta} E_{p,country}} =\frac{\eta_{p,s}}{\eta_{p, country}}= \frac{\phi_{p, s}}{\phi_{p,s, country}} $$

The relative performance ratio can also refer to an emission reduction scenario, and be used, for example, in the analysis of the application of the NEC directive presented in “Case study: analysis of the application of the NEC directive”. Equation 10 is therefore adapted into Eq. 11.

11 $$ \rho_{NEC} = \frac{{\Delta} I_{NEC}}{{\Delta} I_{NEC,country}} $$

where Δ*I*_*NEC*_ represents the variation of the impacts, in terms of average PM_2.5_ exposure change (population-weighted concentration change), due to the implementation of the NEC directive through an emission reduction scenario. The emission reduction scenario is defined by assigning the emission reduction per precursor listed in the directive to specific sectors or areas, using the spatially specific SRR. Δ*I*_*NEC*,*country*_ represents the variation of exposure obtained by applying the same total precursor emission reductions averaged to the whole country, mimicking country-to-grid SRR.

The scenarios designed to assess the impacts of the application of the directive beyond country level can also be evaluated directly in terms of percentage impact reduction Δ*I*_*NEC*_[%], relatively to the base case scenario, represented by the average yearly exposure modelled in CHIMERE considering emissions for the year 2010 and meteorology for the year 2009 (*I*). The percentage exposure reduction is expressed in Eq. 12.

12 $$ {\Delta} I_{NEC} [\%] = \frac{{\Delta} I_{NEC}}{I} \cdot 100 $$

All the indicators defined in this section are calculated for each precursor-sector, for precursor-area combination and for different emission reductions scenarios and presented in “Results: impacts of SRR assumptions on air quality policies”.

## Results: impacts of SRR assumptions on air quality policies

In this section, the impacts of spatially specific SRR are analysed by comparing spatial (Spatial reductions) and sectoral (Sectoral reductions) reductions.

The *precursors* considered are PPM, NO_x_, SO_2_ and NH_3_. NMVOC is not considered here as it does not impact PM_2.5_ concentrations significantly (Pisoni et al. 2017; Amann and Wagner 2014). The *sources* are (i) FUA and non-FUA areas for spatial reductions and (ii) the sectors represented by industry, residential, transport, agriculture and other for sectoral reductions. For ease of visualisation, the *receptor* countries represented here are limited to Belgium (BE), France (FR), Italy (IT), Germany (DE), Spain (ES) and the United Kingdom (UK). Results show that the impact of emission reductions can be overestimated (or underestimated) depending on which sector and where reductions are applied.

### Spatial reductions

For the evaluation of the *spatial emission reductions*, the FUA areas and the non-FUA areas are considered for each country. Table 2 reports the percentage of the area and of the overall population that belongs to FUAs in each country considered. For these countries, the population living in FUAs is generally well over half of the total population while FUAs represent generally much less than half of the surface of the country. This table already indicates that, in order to reduce population exposure, it is necessary to abate pollution in densely populated areas.

**Table 2:** Population and area percentages belonging to urban areas (FUAs), for each country

	Pop [%]	Area [%]
BE	60.05	35.07
FR	70.60	27.82
IT	54.00	18.17
DE	74.14	54.02
ES	66.90	12.38
UK	72.28	26.39

**Fig. 2:**
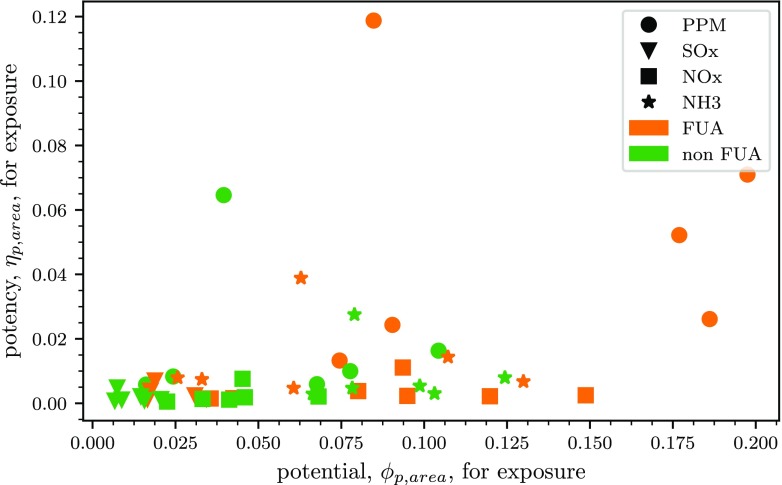
Potency (*μg*/*Gg*) vs potential for average PM_2.5_ exposure, for each precursor reduction in FUAs and elsewhere for all the countries considered (Belgium (BE), France (FR), Italy (IT), Germany (DE), Spain (ES) and the United Kingdom (UK), for visualisation purposes they cannot be distinguished in the figure)

For each precursor reduction implemented in FUAs or elsewhere, the corresponding potencies, *η*_*p*,*s*_, and relative potentials, *ϕ*_*p*,*s*_, are calculated as explained in the methodology (Methodology: impact of SRR spatial detail on AQ policies) and as shown in Eqs. 6 and 8 respectively. All results are summarised in Fig. 2.

The spread of the values displayed in Fig. 2 reflects the different countries considered; however, it is possible to see that, in general, PPM reductions in FUAs correspond to high values of both potency (*η*_*p*,*s*_) and potential (*ϕ*_*p*,*s*_). These high values of potencies reflect the direct contribution of PPM to PM_2.5_ concentrations whereas the high values of potential indicate abundant emissions. For NO_x_ emissions, the potency is low as time is needed to convert the emissions to PM_2.5_ concentrations through secondary PM formation mechanisms. However, despite the low potency, potentials are higher as NO_x_ emissions are generally abundant. For SO_x_, both potency and relative potentials are low. For NH_3_, country per country and for the countries considered here, potencies are higher than those of NO_x_ and SO_x_ but much lower than those of PPM (see Table 4 in the Appendix).

**Fig. 3:**
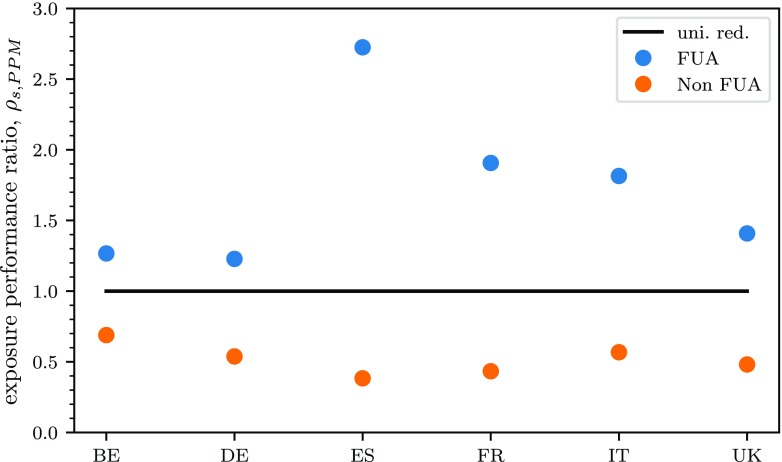
Comparison of the performance ratio (in terms of average PM_2.5_ exposure change), *ρ*_*p*,*s*_, of spatial emission reductions of PPM in FUAs and elsewhere with respect to uniform reductions (uni. red.) over the whole country

For a systematic comparison, the relative performance ratio, *ρ*_*p*,*s*_, evaluates the relative exposure change obtained with emission reductions in FUAs and elsewhere with respect to uniform reductions over the whole country. Results are presented for precursors PPM in Fig. 3. The corresponding figures for the other precursors and for the impacts on concentration can be found in Appendix 1.

As expected, emission reductions in FUAs produce a greater reduction in exposure compared to country and non-FUA reductions. For Spain in particular, PPM emission reductions in FUAs are about 2.5 times more efficient than emission reductions on the whole country. This means that, for Spain, for a kton of PPM reduced in FUAs, the reduction of the overall exposure is 2.5 times the one obtained for a kton of PPM reduced in the whole country (equally distributed, in relative terms, to the emissions of each cell of the country). The results for Germany are however very different, and the potency of PPM is *only* about 1.2 times the potency obtained for whole country reductions.

In order to explain the differences of performance ratios for exposure between different countries, it is useful to look at Table 2. Spain, for example, displays a large (about 67%) share of the population living in FUAs which occupy only 12% of the surface of the country. Emission reductions are therefore highly concentrated in a relatively small area affecting a lot of people. In Germany, on the contrary, a larger share, about 54%, of the surface of the country corresponds to the large share of population living in FUAs (about 74%). The shapes of FUAs for each country considered are graphically presented in Fig. 11 in Appendix 1. In fact, when looking at the impacts on concentration, in Appendix 1, the differences between reductions in FUAs and elsewhere are much smaller, and generally vary between 0.9 and 1.1 times the impacts obtained with uniform country reductions. This is because all cells of the domain are considered equally and are not weighted in terms of population.

### Sectoral reductions

**Fig. 4:**
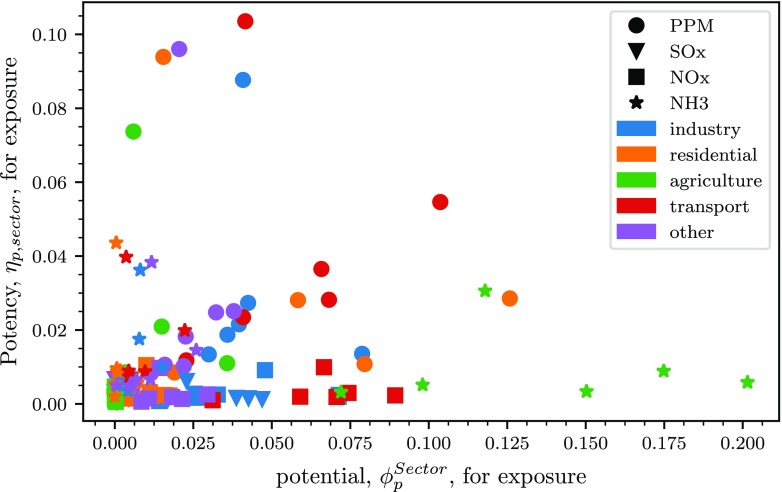
Potency (*μg*/*Gg*) vs potential for average PM_2.5_ exposure, for each sector-precursor combination, for all countries considered (Belgium (BE), France (FR), Italy (IT), Germany (DE), Spain (ES) and the United Kingdom (UK), for visualisation purposes they cannot be distinguished in the figure)

The same approach used to evaluate emission reductions spatially is used to compare *sectoral emission reductions*, for each country and each precursor, to corresponding emission reductions on the overall country. As before, in order to understand the *effectiveness*, towards the reduction of the impacts of each precursor-sector combination, the corresponding potencies, *η*_*p*,*s*_, and relative potentials, *ϕ*_*p*,*s*_, are calculated and the values obtained are summarised in Fig. 4.

This figure shows that even though results are again spread, many sector-precursor combinations have little relevance as they are characterised by both low potency and low potential (at the bottom left corner of the figure). It also shows that NH_3_ emission reductions in agriculture generally have rather low potency but very high potential in reducing PM_2.5_ exposure. This means that even though the reduction of impact per unit reduction of emission is small, the total relative potential for reduction is large. Similarly, NO_x_ emissions from the transport sector generally have a higher potential in reducing PM_2.5_ exposure than NO_x_ emissions from other sectors.

**Fig. 5:**
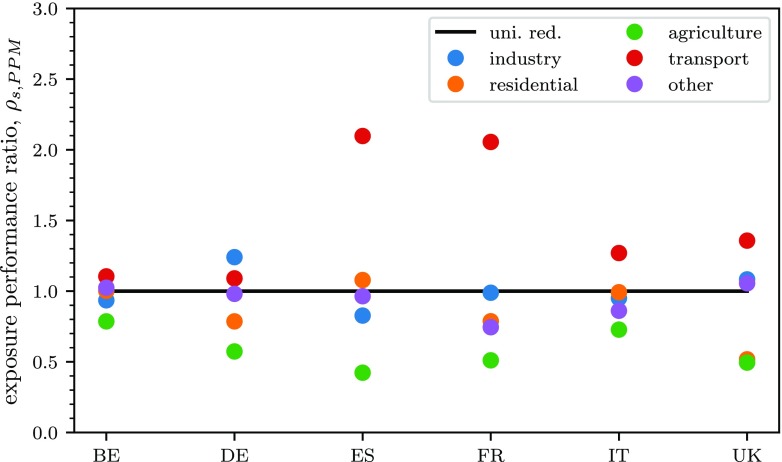
Comparison of the performance ratio (in terms of average PM_2.5_ exposure change), *ρ*_*p*,*s*_, for exposure of the sectoral reductions of PPM with respect to uniform reductions (uni. red.) over the whole country

Results, in terms of relative performance on exposure, are presented in detail for PPM in Fig. 5. As before, the corresponding figures for the other precursors and for the impacts on concentration are reported in Appendix 1.

It is interesting to notice how transport, which inevitably tends to be concentrated where the population lives, always yields reductions in the exposure that are higher than country averaged uniform reductions. The opposite is true for emissions from agriculture, NH_3_ in particular, which are more uniformly distributed across the countries (see Appendix 1). However, it should be kept in mind that, as shown in Fig. 4, agriculture has the highest relative potentials for NH_3_ while relative potentials for the other sectors are much smaller.

As said for the spatial emission reductions, when looking at the impacts on concentration, the differences between reductions on single sectors and the whole country are much smaller but still significant; they generally vary between 0.5 and 1.5 times the impacts obtained with whole country reductions.

## Case study: analysis of the application of the NEC directive

The NEC Directive indicates, for each member states, emission reductions to be achieved by the years 2020 and 2030 with reference to the 2005 baseline. The impact of its implementation beyond the country level is here analysed by considering specific areas and sector-precursor combinations to which to apply the reductions. Impacts are compared to the ones obtained by equivalent uniform emission reductions over the whole country. Results show how the implementation of the NEC directive by member states can affect the outcomes depending on the underlying assumptions.

Table 3 reports the target emission reductions for 2030 considering a baseline the year 2005, as stated in the NEC directive, and the year 2010, as the reference year for the inventory underlying the SRR model used here. The emission reductions relative to 2010 are obtained by considering the emissions reported in the PRIMES 2013 REF-CLE scenarios for the years 2005 and 2010. The NEC directive is applied to the inventory for the year 2005 to obtain the target national total emissions. The reductions relative to 2010 give the same national totals considering the emission scenario for that year.

**Table 3:** NEC directive percentage emission reductions relative to the year 2005 (as reported in the NEC directive) and for the year 2010 according to the PRIMES 2013 REF-CLE scenario, in order to achieve the same emissions targets by 2030

	2005	2010	2005	2010	2005	2010	2005	2010	2005	2010
	PPM	PPM	NO_x_	NO_x_	SO_2_	SO_2_	NH_3_	NH_3_	NMVOC	NMVOC
BE	39	30	59	50	66	30	13	13	35	13
DE	43	39	65	60	58	54	29	26	28	19
ES	50	42	62	32	88	55	16	9	39	23
FR	57	54	69	60	77	61	13	12	52	34
IT	40	51	65	52	71	45	16	5	46	30
UK	46	44	73	63	88	80	16	8	39	18

In this study, different scenarios are built from the commitments reported in the NEC Directive to evaluate emission reductions by sub-country areas and by sector. In this analysis, there are two types of areas, FUAs and non-FUAs, and five different sectors, introduced in Table 1, which have different relative performances in each country. It is therefore necessary to introduce a systematic *prioritisation* of the area-precursor and sector-precursor combinations to which emission reductions are applied. Scenarios are therefore built by applying the emission reduction required by the NEC directive, for each precursor, to sectors or areas according to decreasing (or increasing) values of *η*_*p*,*s*_. These reductions, on a per precursor basis, are within the emission reduction ranges used to train and validate the model. Emissions, for each precursor, are reduced from the area or sector that displays the highest (lowest) value of *η*_*p*,*s*_, to the consecutive ones (for sectors) or to the rest of the country (for areas) until the target emission is achieved. As said earlier, the relative potential, *ϕ*_*p*,*s*_, is also important as it indicates the share of the impact that can be avoided by reducing the corresponding emissions. The same approach could be in principle applied considering the relative potential, instead of the potency, to prioritise the area-precursor and sector-precursor combinations accordingly. However, the prioritisation according to the potencies allows the evaluation of how the reduction of a fixed amount of emissions can be implemented the most or the least effectively. Intrinsically, the sector-precursor or area-precursor combinations *chosen* with priority have a small impact if their relative potential is small.

The values of *η*_*p*,*s*_, normalised to their maximum value are reported in Appendix 3. Emission reductions per precursor, per country, per sector or per area type, resulting from the prioritisation given by increasing or decreasing values of *η*_*p*,*s*_, are also reported in Appendix 3.

Of course, this *prioritisation* of sectors or areas and precursor combinations and the resulting scenarios are *ideal* as measures to improve air quality will likely not completely reduce a single precursor from a single sector or area type before affecting another one. Furthermore, measures usually affect more than one precursor and even more than one sector simultaneously. The aim of this analysis, however, is not to identify the optimal implementation of the NEC directive, but only to quantify the variability of the impacts that can be obtained by considering a spatially specific approach.

Results can therefore be analysed in terms of the percentage *relative impact on exposure*, as shown in Eq. 12 and, again, considering the *relative performance ratio*, as shown in Eq. 11. In the following sections, “Case study: application of the NEC directive at country scale, spatial reductions” and “Case study: application of the NEC directive, sectoral reductions”, results are reported in terms of impacts on exposure.

### Case study: application of the NEC directive at country scale, spatial reductions

From the results reported in “Spatial reductions”, it is possible to infer that emission reductions applied with priority to the precursor-area combinations according to decreasing values of potency are always applied first to FUAs and then to the rest of the country.

**Fig. 6:**
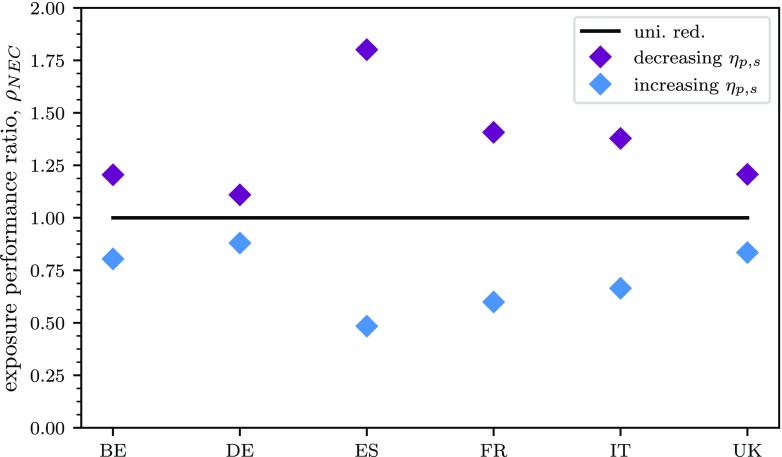
Impact on exposure performance ratio (in terms of average PM_2.5_ exposure change) of the NEC directive considering a prioritisation of areas according to decreasing and increasing values of potency, *η*_*p*,*s*_

Results therefore, as expected, always display the greatest abatement in exposure when emissions are reduced with priority in FUAs, as displayed in Fig. 6.

**Fig. 7:**
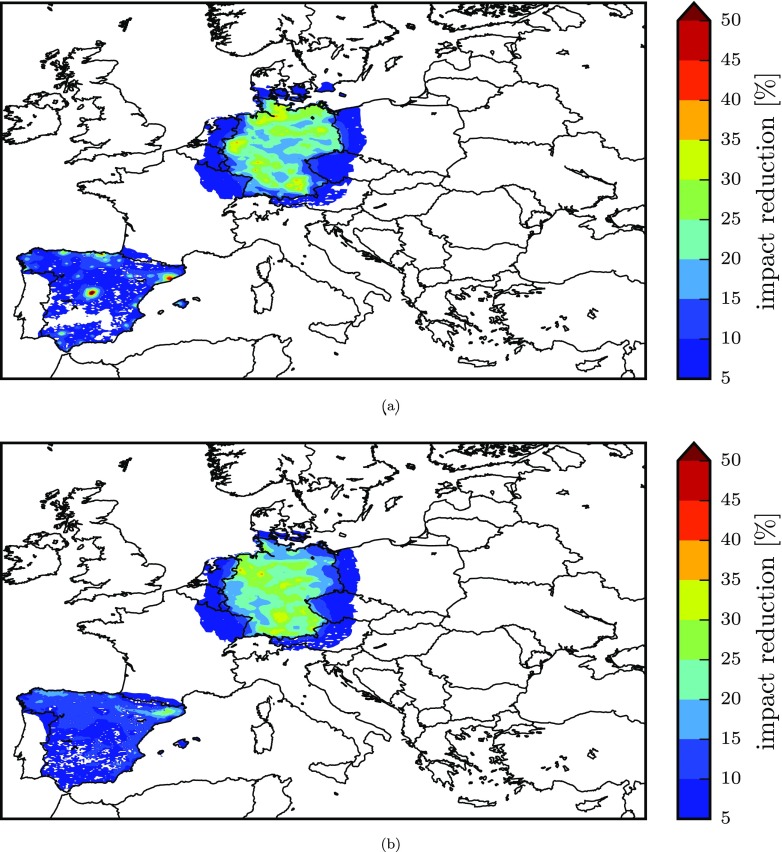
Gridded impact on exposure to PM_2.5_ for spatial reductions in Germany and Spain, corresponding to the results of Fig. 6. Impacts resulting from the prioritisation of areas for decreasing values of potency (*η*_*p*,*s*_) are reported in **a** for increasing ones are reported in **b**

For a spatial representation of the impacts, Fig. 7 displays, for Spain and Germany, the gridded relative percentage impact on exposure resulting from the implementation of the NEC directive with priority to FUA areas (areas with the highest potency) and with priority to non-FUA areas (areas with the lowest potency). This figure shows the effect of the different distribution of FUAs in the two countries. On the one hand, for Germany, the exposure reduction in both cases is similar although differently distributed. On the other hand, for Spain, emission reductions applied to FUAs give a much greater reduction in impacts, especially in the areas of Madrid and Barcelona.

### Case study: application of the NEC directive, sectoral reductions

**Fig. 8:**
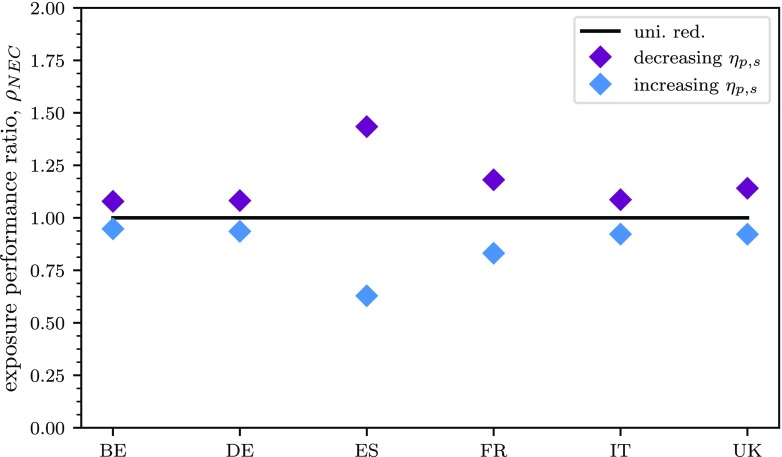
Impact on exposure performance ratio of the NEC directive considering a prioritisation of sectors according to decreasing and increasing values of potency, *η*_*p*,*s*_

Sectoral emission reductions from high to low values of *η*_*p*,*s*_ (prioritisation with decreasing values) yield results always above the equivalent uniform reductions over the country, whereas from low to high values of the same indicator (prioritisation with increasing values) always yield results below the equivalent flat reductions over the country, as shown in Fig. 8. The reduction in exposure obtained with reductions on the prioritised sectors generally vary between 0.75 and 1.25 times the reduction in exposure obtained with uniform emission reductions of a country-to-grid approach.

In terms of sectoral prioritisation, the particular role of agriculture in this case study should be highlighted. Agriculture is penalised because it generally appears last in the priority order set by the most effective prioritisation, from high to low values of potencies, and because the NEC directive generally requires lower percentage reductions of NH_3_ with respect to other precursors. However, one should remember that NH_3_ reductions in agriculture display a relative potential generally higher than that of other sector-precursor combinations (as previously shown in Fig. 4) and can therefore have an important impact in the reduction of exposure.

**Fig. 9:**
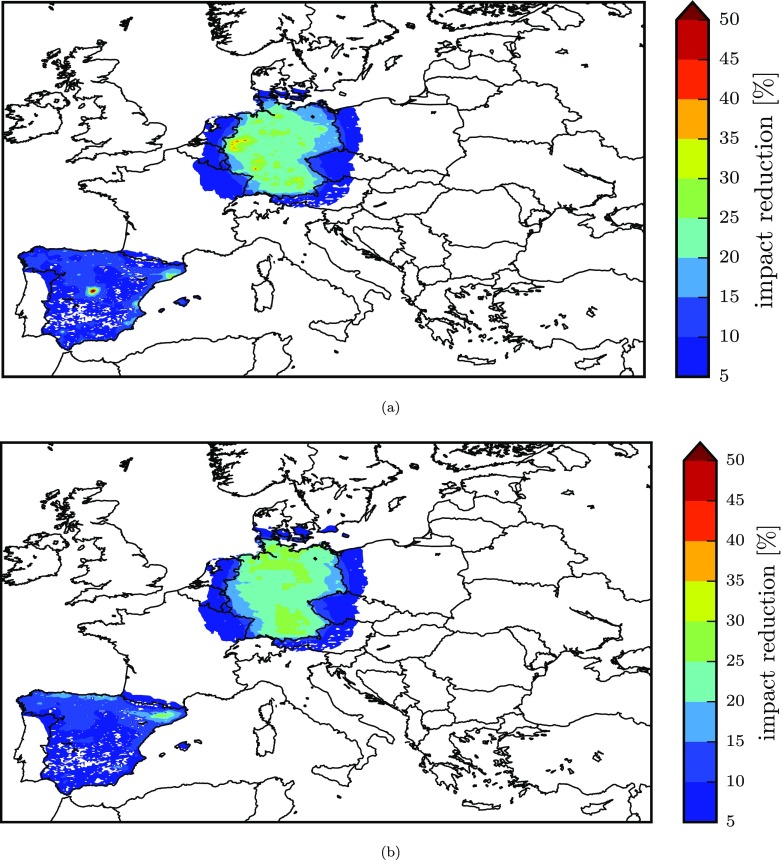
Gridded impact on exposure to PM_2.5_ for sectoral reductions in Germany and Spain, corresponding to the results of Fig. 8. Impacts resulting from the prioritisation of sectors for decreasing values of potency *η*_*p*,*s*_ are reported in **a** for increasing ones are reported in **b**

The gridded impacts resulting from the sectoral prioritisation are represented for Spain (which shows the greatest variability among the countries considered) and for Germany in Fig. 9. This figure also visually shows that emission reductions applied with priority to the precursor-sector combinations that display the highest potency result in greater impact in terms of exposure reduction, highlighting the regions of Madrid and Barcelona in Spain as in the analysis of spatial reductions (2) and, in this case, the Ruhr-Westphalia Industrial Region and Middle Rhine Industrial Region for Germany.

The results obtained with the sector-precursor prioritisation according to their potency represent the *extreme case scenario* in terms of differences between SR approaches applied to different sectors. These results, in fact, display the largest possible differences between the reduction in exposure obtained for the application of the NEC directive with the spatially specific SRR approach and the country-to-grid one.

### Case study: application of the NEC directive, impact on cities

**Fig. 10:**
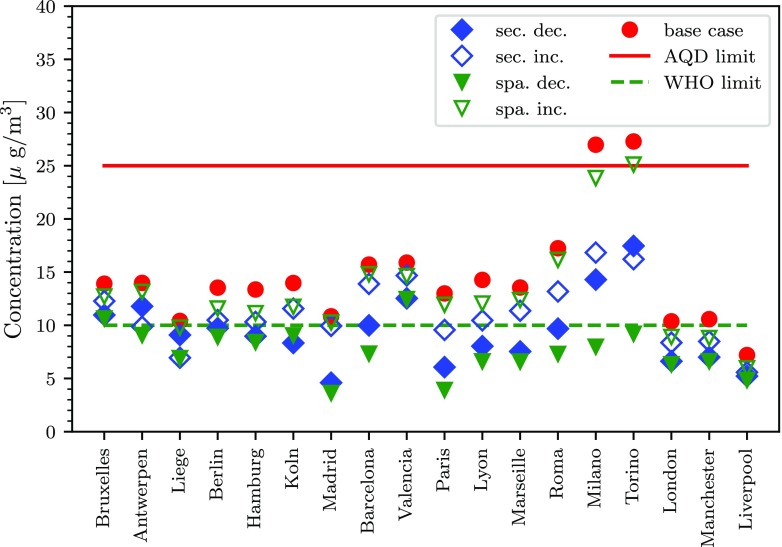
Urban background PM_2.5_ concentrations for the base case and for the scenarios analysed in this study: sectoral (sec.) reductions and and spatial (spa.) reductions applied giving priority to combinations with decreasing (dec.) or increasing (inc.) values of *η*_*p*.*s*_. The limit concentration value according to the AQD (25 *μ* g/m^3^ annual mean) and the WHO guidelines (10 *μ* g/m^3^ annual mean) are also reported as a reference. Values are scaled to the measured PM_2.5_ concentration in each FUA, obtained between 2011 and 2016 (European Environment Agency (EEA) 2017)

Results of the analysis carried out in this study highlight the spatial variability of the impacts on exposure, and therefore on health that emission reductions of the same precursor can have when applied to different sectors and areas. The different distributions of percentage exposure reduction obtained in Figs. 7 and 9 underline the importance of the integration of national and sub-national policies to address AQ. To further underline the issue, Fig. 10 displays the estimated urban background PM_2.5_ concentration obtained for each scenario analysed in this study, for three major cities of each country. The base case values are represented by the average urban background PM_2.5_ measurements obtained between 2011 and 2016 in the FUA of each city. The concentration values are available in the Air Quality e-Reporting tool provided by the European Environment Agency (EEA) (European Environment Agency (EEA) 2017). The relative concentration reduction modelled in the target cell corresponding to the centroid of each FUA is applied to the base case to obtain the scaled concentration of each scenario. As a reference, the average yearly PM_2.5_ concentration limits according to the AQD of 25 *μ* g/m^3^ and the more stringent one of 10 *μ* g/m^3^ advised by the World Health Organization (WHO) (World Health Organization 2016) are also reported.

Figure 10 shows that even though most of the European cities *comply*^1^ with the AQD, the application of the NEC directive under the scenarios considered in this study is often not sufficient to achieve urban background PM_2.5_ concentration levels below the ones advised by the WHO. As can be expected, the application of the NEC directive to spatial reductions with the highest potencies that is with priority to FUAs generally allows to achieve the lowest PM_2.5_ concentration levels. Sectoral emission reductions applied at the national level can display, in certain cities, very similar performance to emission reductions applied spatially. For example, for Valencia, sectoral and spatial reductions display very similar results. As expected, in most cases, emission reductions applied with priority to sectors with decreasing values of *η*_*p*,*s*_ result in lower concentrations than when applied in reverse order (with priority for increasing values of *η*_*p*,*s*_). Certain cities, for example Torino, do not display the same trend. This is because the *ideal* sectoral prioritisation according to decreasing values of *η*_*p*,*s*_ at the local level can be different to the one defined at the national level. Furthermore, there can be important differences between large cities within a same country, as is the case for Italy with Milano and Torino with respect to Roma. To summarise, the results displayed in Fig. 10 show the impact, at the local level, of sectoral and spatial emission reductions applied at the national level. Differences between cities highlight the importance of taking into consideration local conditions. Further reductions in the concentration levels could be obtained by combining the sectoral and spatial prioritisation accounting for local specificity.

### Case study: application of the NEC directive, discussion

The analysis of the application of the NEC directive is here considered as a *conceptual* case study to evaluate through *ideal* emission reductions scenarios the effects of the assumptions behind the SRR. The analysis of a *realistic* implementation of the NEC directive, by sector on the overall country, using the spatially specific SRR will always yield a *relative impact* and a *relative performance* within the bounds set by the *decreasing* and *increasing* priority order given by *η*_*p*,*s*_ obtained in the analysis of the sectoral reductions (2).

Equivalently, the implementation of the NEC directive by area type, will yield results within the bounds obtained in the analysis of the spatial reductions (2).

It is important to underline that each member state implements the directive according to other priorities, such as feasibility and costs, as well as other factors, which are not considered here. Costs, in particular, will affect the priority of precursor emission reductions. For example, if the costs of measures reducing NH_3_ (from the agricultural sector) were lower than the costs of additional NO_x_ or SO_2_ measures (in other sectors), emission reductions from agriculture would be prioritised.

Furthermore, the evaluation of the impacts is limited to only PM_2.5_, whereas the reduction of precursors also impacts NO_2_ and O_3_ (ozone) exposure, which are also detrimental to human health albeit with a lower risk rate (World Health Organization Europe 2013).

Finally, the results presented in this section underline the importance of AQP which are regionally and sectorally specific.

## Discussion

This study analyses the effects of using spatially specific SRRs to evaluate emission reductions. The analysis shows that there can be strong differences, in terms of resulting exposure and, to a lesser extent, concentration changes, when the same precursor emission reductions are applied to specific areas and sectors. This, in turn, can impact the *expected* outcome of policies on air quality.

### Limitations and further developments

The limitations of this study include the uncertainty of the inventory, of the underlying CTM and of the SRR modelling approach and that exposure to only one pollutant (PM_2.5_) is considered. The urban background PM_2.5_ concentration is evaluated for only one meteorological year (2009) and the resolution is constrained to a ∼ 7 × 7 km^2^ grid.

Furthermore, as said earlier, the analysis of the NEC directive is here considered as a *conceptual* case study and the emission reduction scenarios by sector and areas are to be considered *ideal*. Therefore, this study does not aim to provide the *optimal* implementation of the NEC directive nor to review its targets.

Further developments of this study should take into account, apart from the sectoral and spatial dimensions of emission reductions, also specific measures, which can simultaneously reduce more than one precursor, and their costs. The impacts should be extended to include NO_2_ and O_3_ (ozone) exposure with a complete health impact assessment. The current analysis considers only impacts of emission reductions within each country on itself. It would therefore be interesting to extend the analysis at EU level and consider transboundary effects.

### Conclusions

The analysis of the effect of emission reductions for different precursor-sector combinations shows, for example, that the reduction of emissions in the transport sector yields impacts, per unit of emissions, higher than uniform percentage reductions over the whole country. This is of course because transport emissions are generally close to the population. The opposite is true for agriculture, as the emissions are more uniformly distributed. For other sectors, it depends on the country. The study of the effects of emission reductions in the FUAs with respect to elsewhere, consistently shows, as can be expected, that emission reductions in FUAs always yield greater relative impacts, per unit of emission reduced, than country or non-FUA ones. This shows that, for a given precursor, the effect of its emission reductions can be underestimated or overestimated, depending on which sector and where the reductions are applied. As a case study, the analysis is applied to the emission reductions to which member states committed to in the new NEC directive. Results, considering different precursor-sector and precursor-area combinations, allow to quantify the maximum range of variability of the impacts. That is, the extreme maximum and the extreme minimum exposure reductions that can be obtained by applying to different sectors and areas (FUAs and non-FUAs) a given reduction of precursors.

Results highlight on the one hand the importance of the integration of national and sub-national policies and on the other, that in order to maximise positive impacts, it is important to act at a higher level of detail regarding sectors and areas of application. A spatially specific SRR approach allows to take into account these aspects and can therefore be a useful support for national and local authorities for the design and implementation of effective emission reduction strategies in order to both comply with the NEC directive at national level and address AQ issues at local level.

## Electronic supplementary material

Below is the link to the electronic supplementary material.

(PDF 2.14 MB)

## Footnotes

1 It should be underlined that this is a result of averaging measurements values; it is not designed for checking compliance.
