# Supplementary material for: Multi-level policies for air quality: implications of national and sub-national emission reductions on population exposure
Source: Air Qual Atmos Health. 2018 Sep 6;11(9):1121–35. doi: 10.1007/s11869-018-0613-1 (PMC6209043; doi:10.1007/s11869-018-0613-1)
Supplement: Supplementary file 1 — (PDF 2.14 MB) [file 11869_2018_613_MOESM1_ESM.pdf]

A Supporting information

The supporting information includes all complementary figures and tables relative to the results presented in the main text. A.1 reports the impacts of spatial reductions in terms of concentration and exposure for each precursor considered. Equivalently, A.2 reports the impacts of sectoral reductions in terms of concentration and exposure for each precursor considered. It should be underlined, once again, that information relative to the *potency* of emission reductions in a sector or area, should be considered along with its *potential*. For example a sector-precursor combination which displays a high *potency* is of little significance if its *potential* is low. The normalised relative potentials, in terms of exposure, for spatial and sectoral reductions, are reported in Table 11 and Table 7 respectively in A.3. This last section reports all the supporting information used to define ideal scenarios for the application of the NEC directive.

A.1 Spatial reductions

Fig. 11: Percentage of grid cells belonging to the FUAs for each country considered.

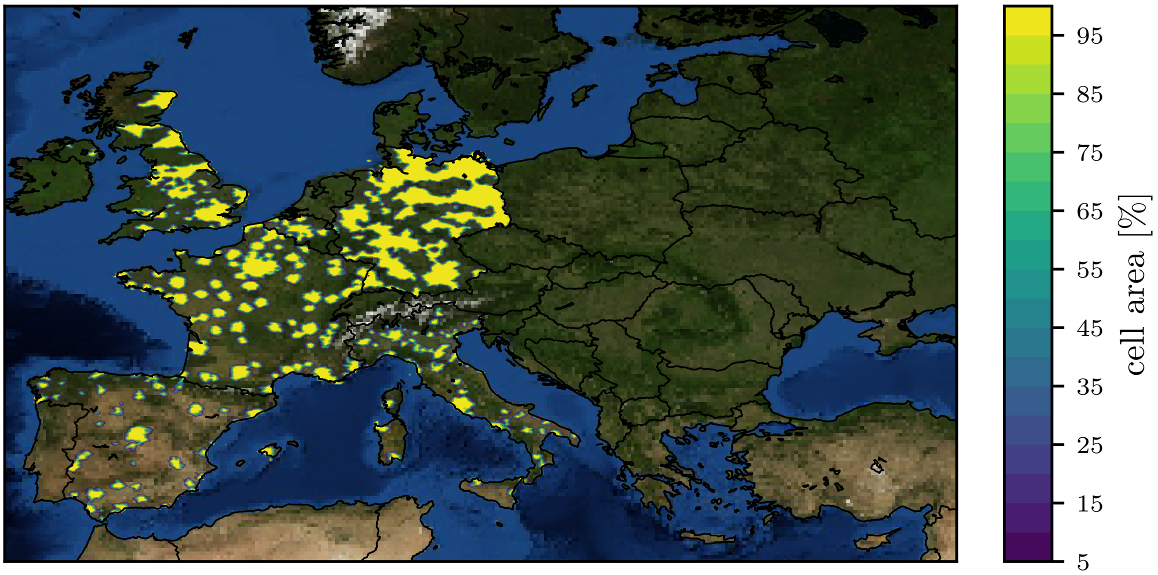

Fig. 12: Spatial reductions: comparison of the performance ratio for exposure to $PM_{2.5}$ due to emission reductions of PPM in FUAs and elsewhere with respect to uniform reductions (uni. red.) over the whole country.

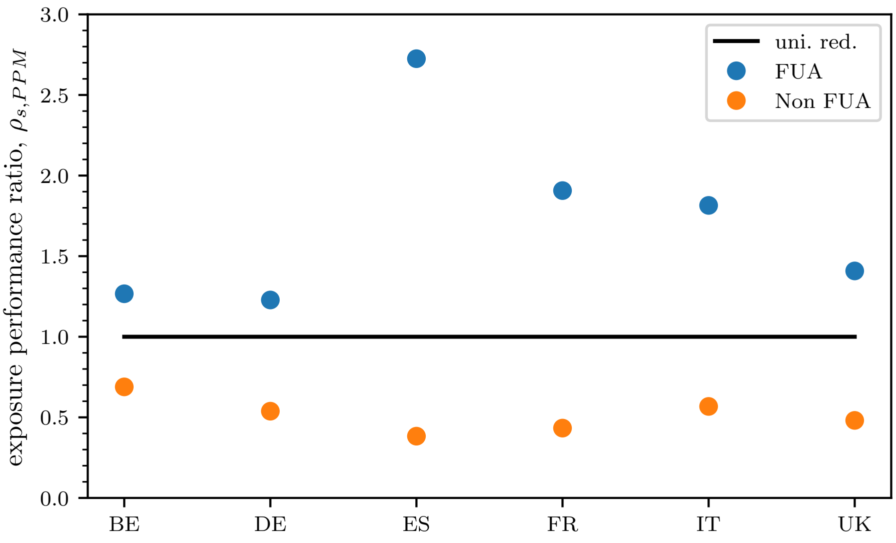

Fig. 13: Spatial reductions: comparison of the performance ratio for exposure to $PM_{2.5}$ due to emission reductions of NOx in FUAs and elsewhere with respect to uniform reductions (uni. red.) over the whole country.

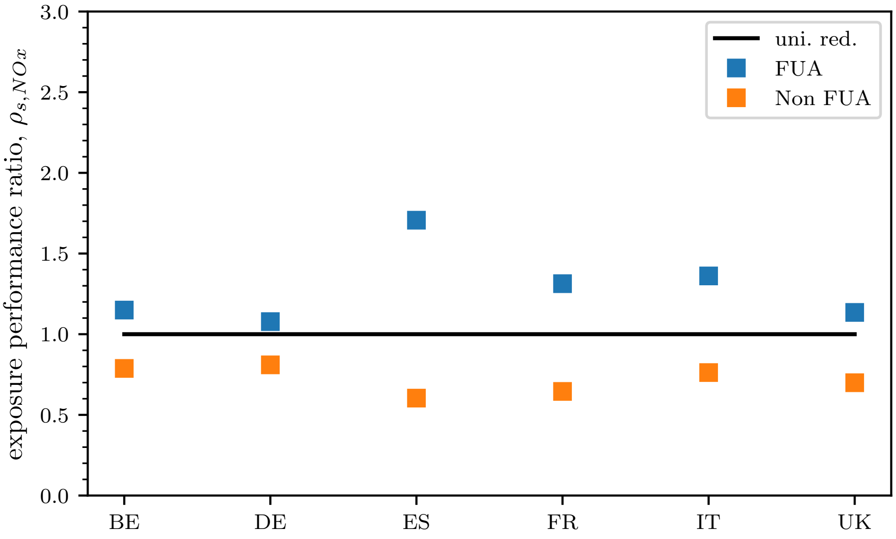

Fig. 14: Spatial reductions: comparison of the performance ratio for exposure to $PM_{2.5}$ due to emission reductions of NH3 in FUAs and elsewhere with respect to uniform reductions (uni. red.) over the whole country.

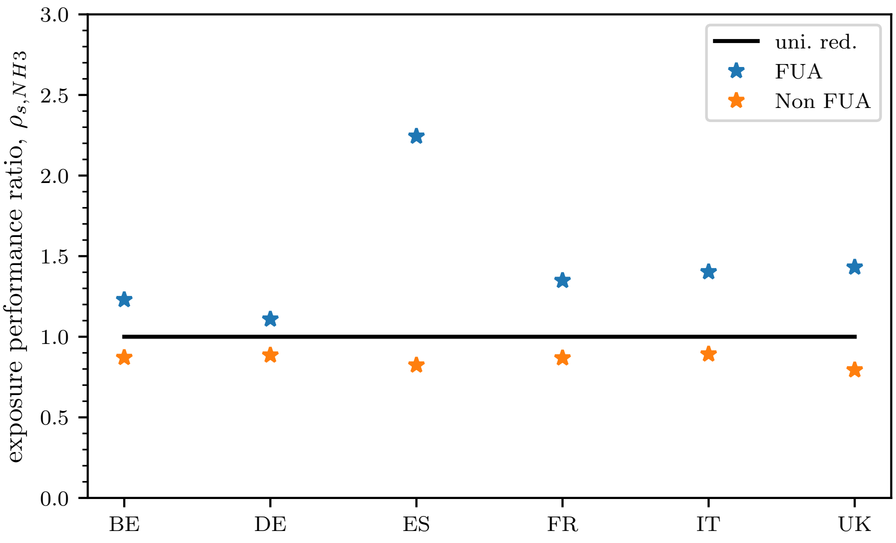

Fig. 15: Spatial reductions: comparison of the performance ratio for exposure to $PM_{2.5}$ due to emission reductions of SOx in FUAs and elsewhere with respect to uniform reductions (uni. red.) over the whole country.

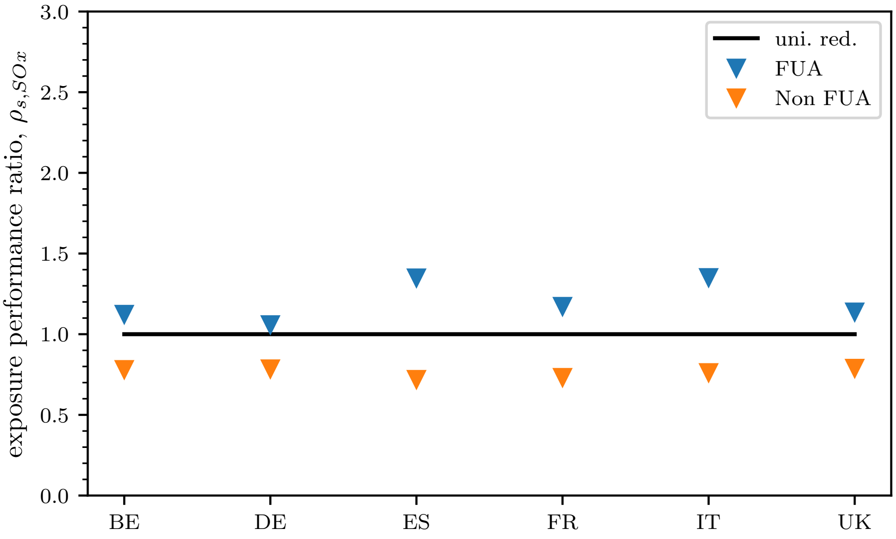

Fig. 16: Spatial reductions: comparison of the performance ratio for concentration of $PM_{2.5}$ due to emission reductions of PPM in FUAs and elsewhere with respect to uniform reductions (uni. red.) over the whole country.

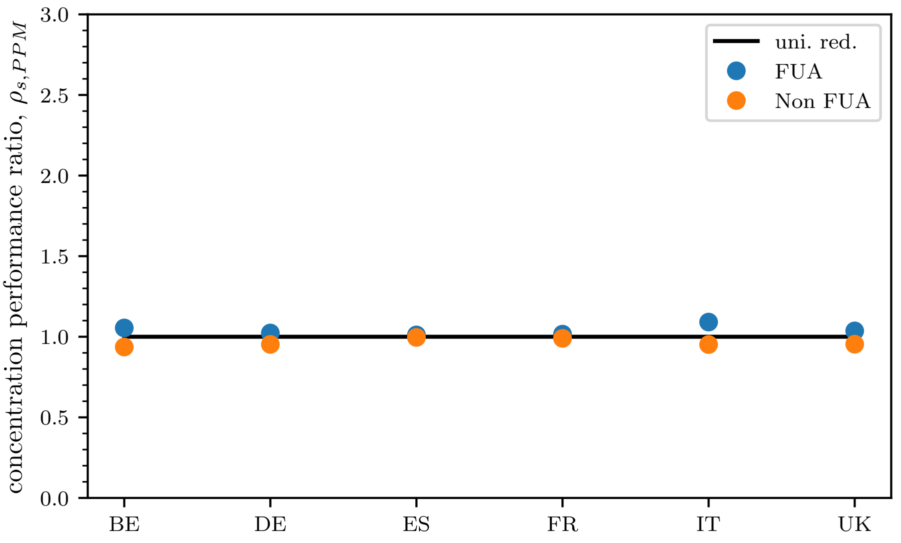

Fig. 17: Spatial reductions: comparison of the performance ratio for concentration of $PM_{2.5}$ due to emission reductions of NOx in FUAs and elsewhere with respect to uniform reductions (uni. red.) over the whole country.

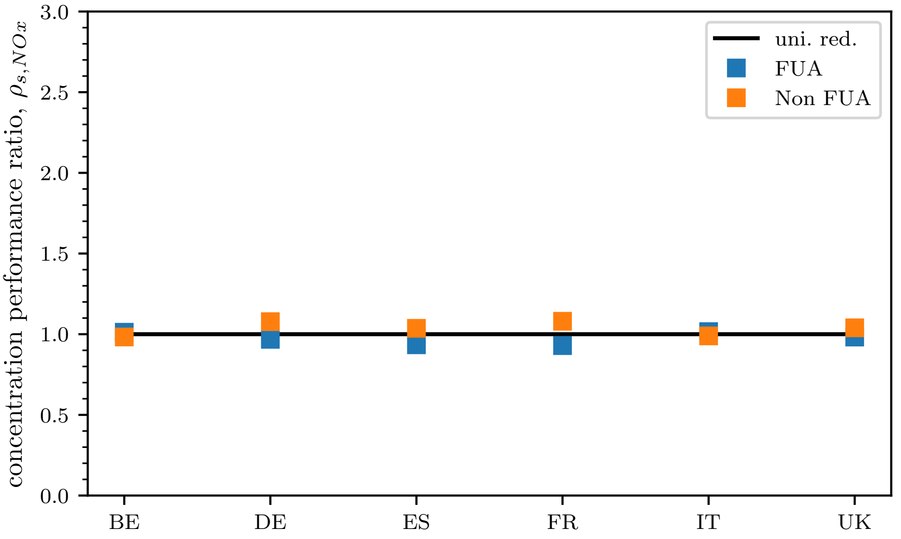

Fig. 18: Spatial reductions: comparison of the performance ratio for concentration of $PM_{2.5}$ due to emission reductions of NH3 in FUAs and elsewhere with respect to uniform reductions (uni. red.) over the whole country.

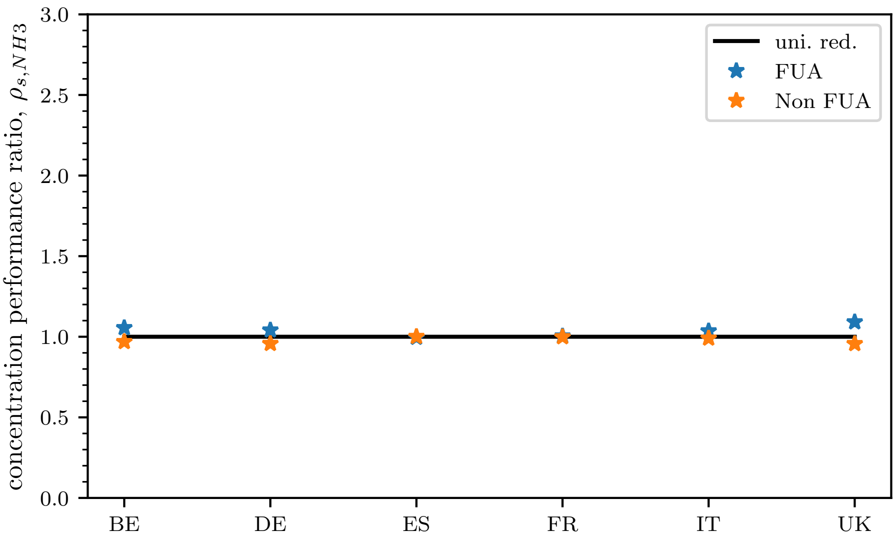

Fig. 19: Spatial reductions: comparison of the performance ratio for concentration of $PM_{2.5}$ due to emission reductions of SOx in FUAs and elsewhere with respect to uniform reductions (uni. red.) over the whole country.

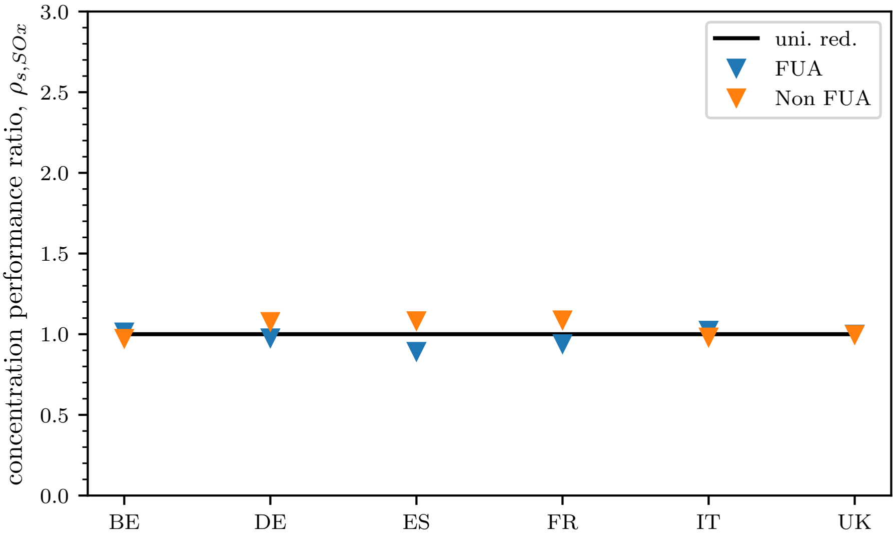

A.2 Sectoral reductions figures

Fig. 20: Sectoral reductions: comparison of the performance ratio for exposure to $PM_{2.5}$ due to sectoral emission reductions of PPM with respect to uniform reductions (uni. red.) over the whole country.

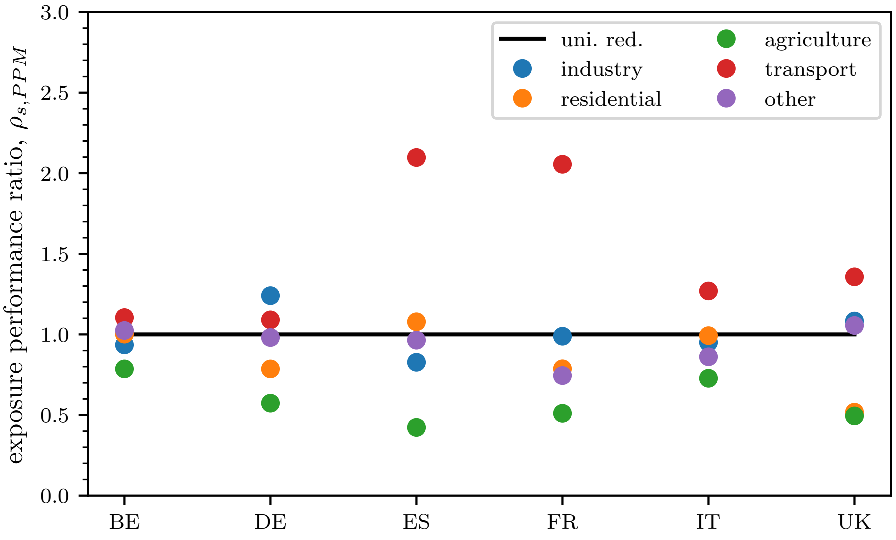

Fig. 21: Sectoral reductions: comparison of the performance ratio for exposure to $PM_{2.5}$ due to sectoral emission reductions of NOx with respect to uniform reductions (uni. red.) over the whole country.

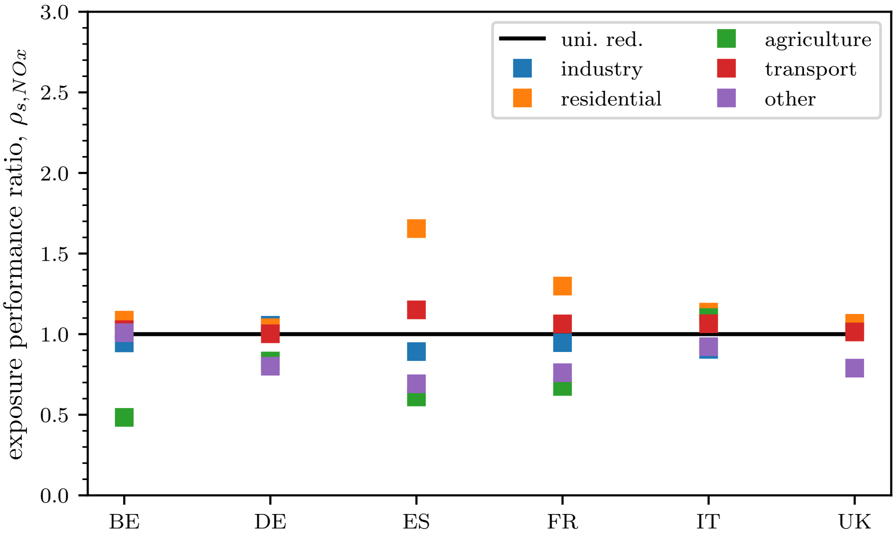

Fig. 22: Sectoral reductions: comparison of the performance ratio for exposure to $PM_{2.5}$ due to sectoral emission reductions of NH3 with respect to uniform reductions (uni. red.) over the whole country.

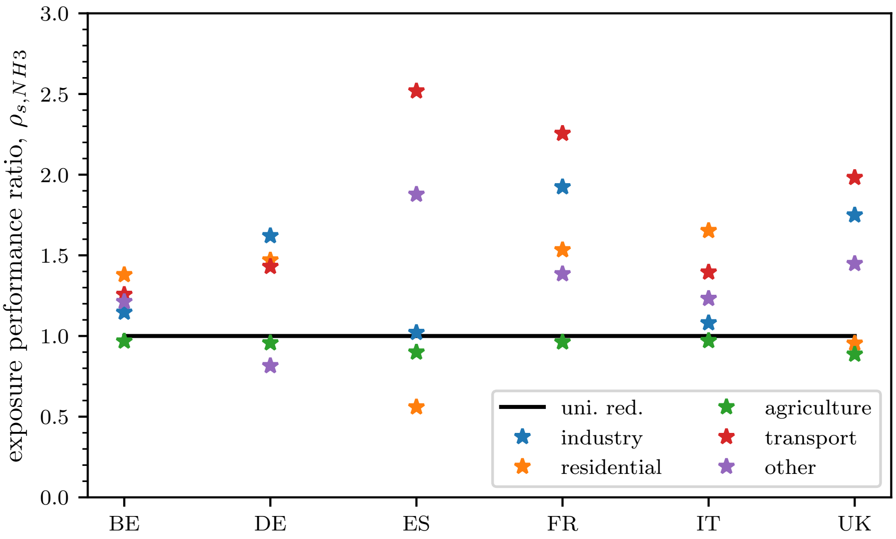

Fig. 23: Sectoral reductions: comparison of the performance ratio for exposure to $PM_{2.5}$ due to sectoral emission reductions of SOx with respect to uniform reductions (uni. red.) over the whole country.

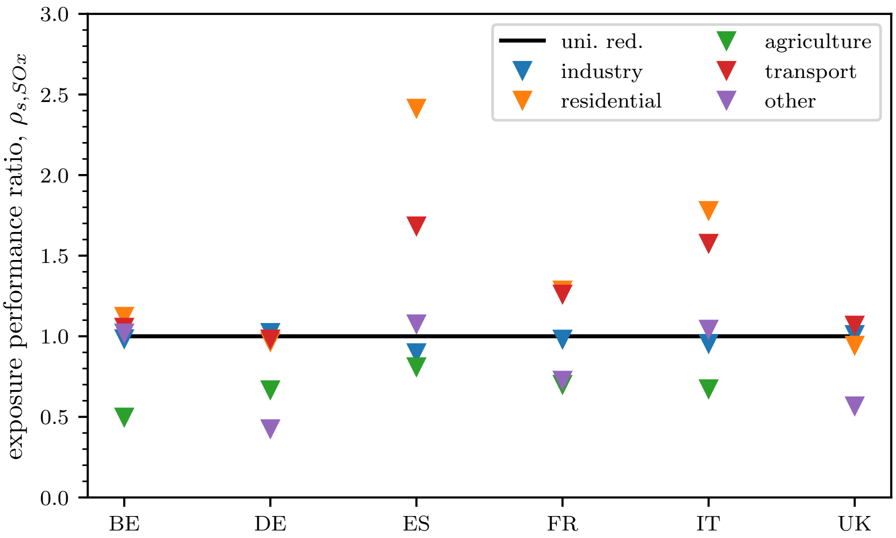

Fig. 24: Sectoral reductions: comparison of the performance ratio for concentration of $PM_{2.5}$ due to sectoral emission reductions of PPM with respect to uniform reductions (uni. red.) over the whole country.

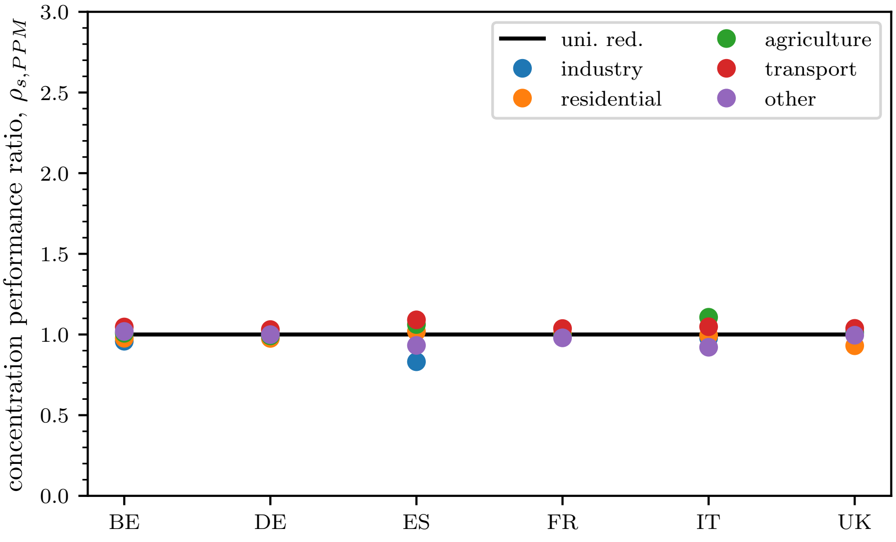

Fig. 25: Sectoral reductions: comparison of the performance ratio for concentration of $PM_{2.5}$ due to sectoral emission reductions of NOx with respect to uniform reductions (uni. red.) over the whole country.

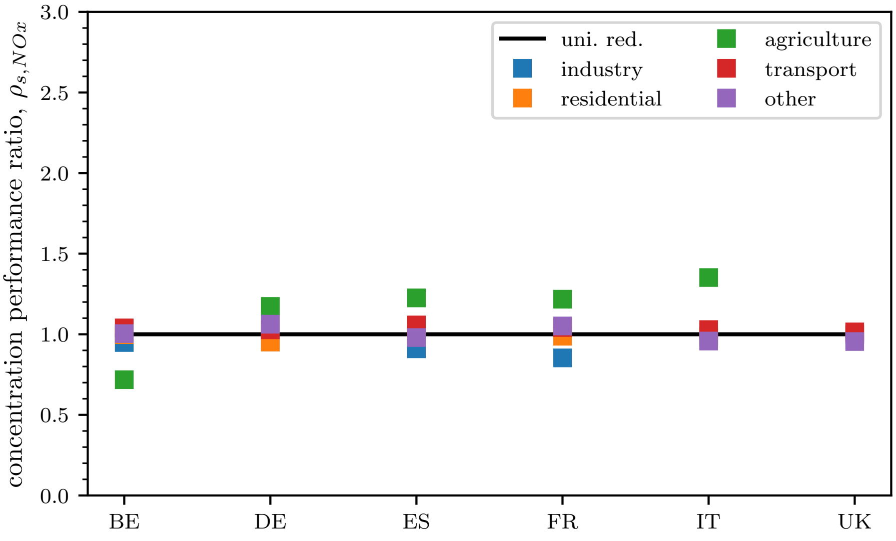

Fig. 26: Sectoral reductions: comparison of the performance ratio for concentration of $PM_{2.5}$ due to sectoral emission reductions of NH3 with respect to uniform reductions (uni. red.) over the whole country.

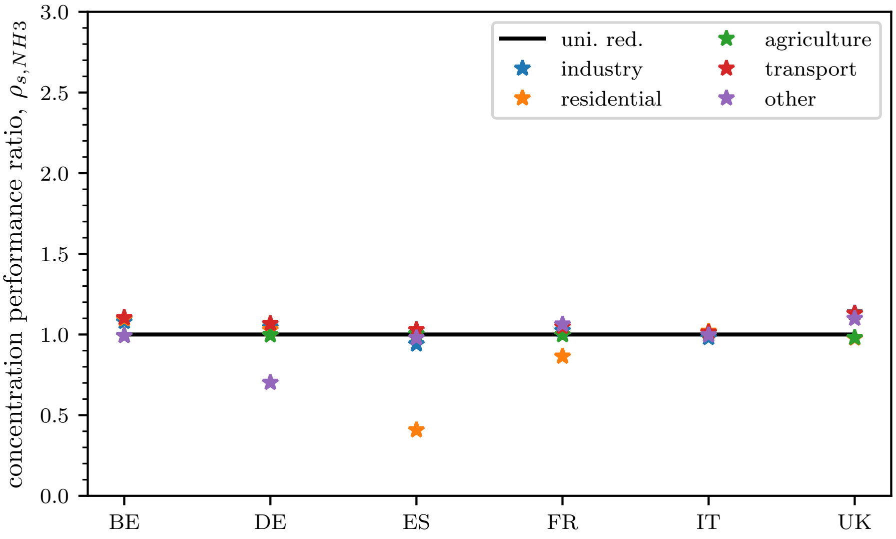

Fig. 27: Sectoral reductions: comparison of the performance ratio for concentration of $PM_{2.5}$ due to sectoral emission reductions of SOx with respect to uniform reductions (uni. red.) over the whole country.

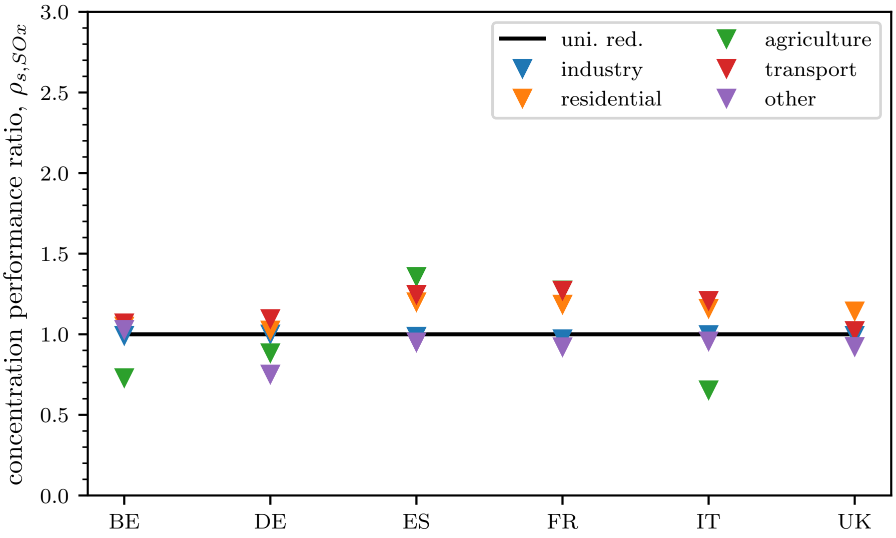

A.3 Supporting information for the NEC directive analysis

Table 4: Potency values for FUA and non FUA for each precursor, as used to prioritise the areas. For ease of interpretation, potency values for each country (in the first two columns) are normalised to the maximum potency of the corresponding area-precursor combination ($\eta_{p,s}/\max(\eta_{p,s})$). The third column reports the maximum value of potency of the area-precursor combination ($\max(\eta_{p,s})$

PPM	FUA	Non FUA	$\max(\eta_{p,s})$ $[ng/m^3/Gg]$
BE	1.00	0.54	118.76
FR	1.00	0.23	26.14
IT	1.00	0.31	52.19
DE	1.00	0.44	13.27
ES	1.00	0.14	70.97
UK	1.00	0.34	24.34
NO_x	FUA	Non FUA	$\max(\eta_{p,s})$ $[ng/m^3/Gg]$
BE	1.00	0.69	11.10
FR	1.00	0.49	2.31
IT	1.00	0.56	3.79
DE	1.00	0.75	2.49
ES	1.00	0.35	1.51
UK	1.00	0.62	2.19
SO_2	FUA	Non FUA	$\max(\eta_{p,s})$ $[ng/m^3/Gg]$
BE	1.00	0.70	7.07
FR	1.00	0.62	1.35
IT	1.00	0.56	3.88
DE	1.00	0.74	1.59
ES	1.00	0.53	2.47
UK	1.00	0.69	1.45
NH_3	FUA	Non FUA	$\max(\eta_{p,s})$ $[ng/m^3/Gg]$
BE	1.00	0.71	38.86
FR	1.00	0.64	4.72
IT	1.00	0.64	7.44
DE	1.00	0.80	6.76
ES	1.00	0.37	7.94
UK	1.00	0.55	14.36

Table 5: Percentage emission reductions per precursor, area and country considering the prioritisation given by decreasing values of $\eta_{p,s}$.

PPM	FUA	Non FUA
BE	60.54	0.00
FR	100	23.04
IT	100	28.37
DE	54.35	0.00
ES	100	20.44
UK	82.42	0.00
NO_x	FUA	Non FUA
BE	83.98	0.00
FR	100	10.21
IT	100	20.01
DE	82.04	0.00
ES	86.67	0.00
UK	100	3.53
SO_2	FUA	Non FUA
BE	44.60	0.00
FR	92.75	0.00
IT	100	10.09
DE	66.87	0.00
ES	100	14.23
UK	100	56.96
NH_3	FUA	Non FUA
BE	34.44	0.00
FR	42.55	0.00
IT	24.62	0.00
DE	50.68	0.00
ES	72.64	0.00
UK	23.57	0.00

Table 6: Percentage emission reductions per precursor, area and country considering the prioritisation given by increasing values of $\eta_{p,s}$.

PPM	FUA	Non FUA
BE	0.00	70.59
FR	0.00	85.54
IT	0.00	81.42
DE	4.92	100
ES	0.00	56.19
UK	3.71	100
NO_x	FUA	Non FUA
BE	13.44	100
FR	20.70	100
IT	0.00	85.84
DE	40.95	100
ES	0.00	48.56
UK	56.66	100
SO_x	FUA	Non FUA
BE	0.00	78.57
FR	27.92	100
IT	0.00	77.59
DE	39.00	100
ES	0.00	94.01
UK	71.71	100
NH_3	FUA	Non FUA
BE	0.00	19.40
FR	0.00	16.11
IT	0.00	6.60
DE	0.00	53.31
ES	0.00	10.30
UK	0.00	11.27

Table 7: Relative potential for FUA and non FUA for each precursor. For ease of interpretation, relative potential values for each country are normalised to the maximum value of the corresponding area-precursor combination ($\phi_{p,s}/max(\phi_{p,s})$). The last column reports the maximum value of relative potential (in percentage) of the area-precursor combination $max(\phi_{p,s})$.

PPM	FUA	Non FUA	max($\phi_{p,s}$) [%]
BE	1.00	0.47	8.48
FR	1.00	0.36	18.61
IT	1.00	0.59	17.69
DE	1.00	0.22	7.45
ES	1.00	0.39	19.75
UK	1.00	0.27	9.04
NO_x	FUA	Non FUA	max($\phi_{p,s}$) [%]
BE	1.00	0.48	9.36
FR	1.00	0.43	9.49
IT	1.00	0.85	8.01
DE	1.00	0.31	14.87
ES	1.00	0.63	3.56
UK	1.00	0.28	11.98
SO_x	FUA	Non FUA	max($\phi_{p,s}$) [%]
BE	1.00	0.39	1.88
FR	1.00	0.40	1.65
IT	1.00	0.84	1.74
DE	1.00	0.21	4.25
ES	1.00	0.67	3.09
UK	1.00	0.46	3.43
NH_3	FUA	Non FUA	max($\phi_{p,s}$) [%]
BE	0.80	1.00	7.90
FR	0.59	1.00	10.31
IT	0.42	1.00	7.83
DE	1.00	0.76	12.99
ES	0.39	1.00	6.64
UK	0.86	1.00	12.44

Table 8: Potency values for each sector and for each precursor, as used to prioritise the sectors. For ease of interpretation, potency values for each country are normalised to the maximum potency of the corresponding sector-precursor combination ($\eta_{p,s}/\max(\eta_{p,s})$). The last column reports the maximum value of potency of the sector-precursor combination $\max(\eta_{p,s})$.

PPM	industry	residential	agriculture	transport	other	$\max(\eta_{p,s})$ $[ng/m^3/Gg]$
BE	0.85	0.91	0.71	1.00	0.93	103.55
FR	0.48	0.38	0.25	1.00	0.36	28.18
IT	0.75	0.78	0.57	1.00	0.68	36.51
DE	1.00	0.63	0.46	0.88	0.79	13.41
ES	0.39	0.51	0.20	1.00	0.46	54.63
UK	0.80	0.38	0.36	1.00	0.78	23.46
NO_x	industry	residential	agriculture	transport	other	$\max(\eta_{p,s})$ $[ng/m^3/Gg]$
BE	0.87	1.00	0.44	0.95	0.93	10.50
FR	0.73	1.00	0.52	0.82	0.59	2.29
IT	0.80	1.00	0.97	0.94	0.81	3.16
DE	1.00	0.99	0.79	0.95	0.76	2.43
ES	0.54	1.00	0.37	0.70	0.42	1.46
UK	0.96	1.00	-	0.95	0.74	2.06
SO_x	industry	residential	agriculture	transport	other	$\max(\eta_{p,s})$ $[ng/m^3/Gg]$
BE	0.88	1.00	0.44	0.94	0.91	7.07
FR	0.76	1.00	0.55	0.98	0.57	1.49
IT	0.54	1.00	0.38	0.89	0.59	5.11
DE	1.00	0.94	0.65	0.96	0.42	1.53
ES	0.37	1.00	0.34	0.70	0.45	4.42
UK	0.95	0.88	nan	1.00	0.53	1.36
NH_3	industry	residential	agriculture	transport	other	$\max(\eta_{p,s})$ $[ng/m^3/Gg]$
BE	0.83	1.00	0.70	0.91	0.88	43.60
FR	0.85	0.68	0.43	1.00	0.61	7.88
IT	0.65	1.00	0.59	0.84	0.75	8.76
DE	1.00	0.91	0.59	0.88	0.50	9.88
ES	0.41	0.22	0.36	1.00	0.75	8.92
UK	0.88	0.48	0.45	1.00	0.73	19.88

Table 9: Percentage emission reductions per precursor, sector and country considering the prioritisation given by decreasing value of $\eta_{p,s}$.

PPM	industry	residential	agriculture	transport	other
BE	0.00	0.00	0.00	100	14.37
FR	100	20.51	0.00	100	0.00
IT	0.00	77.14	0.00	100	0.00
DE	100	0.00	0.00	42.26	0.00
ES	0.00	100	0.00	100	26.52
UK	68.96	0.00	0.00	100	0.00
NO_x	industry	residential	agriculture	transport	other
BE	0.00	100	0.00	91.16	0.00
FR	0.00	100	0.00	98.45	0.00
IT	0.00	100	100	95.18	0.00
DE	100	100	0.00	34.20	0.00
ES	0.00	100	0.00	57.75	0.00
UK	100	100	0	45.29	0.00
SO_x	industry	residential	agriculture	transport	other
BE	19.14	100	0.00	100	100
FR	55.96	100	0.00	100	0.00
IT	35.21	100	0.00	100	100
DE	64.58	0.00	0.00	0.00	0.00
ES	44.11	100	0.00	100	100
UK	89.12	0.00	0	100	0.00
NH_3	industry	residential	agriculture	transport	other
BE	67.36	100	0.00	100	100
FR	100	100	7.38	100	100
IT	0.00	100	0.00	100	36.80
DE	100	100	20.00	100	0.00
ES	11.91	0.00	0.00	100	100
UK	100	0.00	0.00	100	10.82

Table 10: Percentage emission reductions per precursor, sector and country considering the prioritisation given by increasing values of $\eta_{p,s}$.

PPM	industry	residential	agriculture	transport	other
BE	75.51	0.00	100	0.00	0.00
FR	0.00	92.75	100	0.00	100
IT	100	37.06	100	0.00	100
DE	0.00	100	100	0.00	22.21
ES	61.37	0.00	100	0.00	0.00
UK	4.20	100	100	0.00	100
NO_x	industry	residential	agriculture	transport	other
BE	100	0.00	100	5.63	100
FR	100	0.00	100	33.92	100
IT	100	0.00	0.00	12.30	100
DE	0.00	8.37	100	100	100
ES	33.69	0.00	100	0.00	100
UK	50.26	0.00	0	100	100
SO_2	industry	residential	agriculture	transport	other
BE	32.15	0.00	100	0.00	0.00
FR	61.10	0.00	100	0.00	100
IT	55.92	0.00	100	0.00	0.00
DE	41.11	100	100	100	100
ES	61.43	0.00	100	0.00	0.00
UK	81.80	100	0	0.00	100
NH_3	industry	residential	agriculture	transport	other
BE	0.00	0.00	14.44	0.00	0.00
FR	0.00	0.00	12.25	0.00	0.00
IT	0.00	0.00	5.73	0.00	0.00
DE	0.00	0.00	27.88	0.00	100
ES	0.00	100	10.36	0.00	0.00
UK	0.00	0.00	8.95	0.00	0.00

Table 11: Relative potential for each sector and for each precursor. For ease of interpretation, relative potential values for each country are normalised to the maximum value of the corresponding sector-precursor combination ($\phi_{p,s}/max(\phi_{p,s})$). The last column reports the maximum value of relative potential (in percentage) of the sector-precursor combination $max(\phi_{p,s})$.

PPM	industry	residential	agriculture	transport	other	$max(\phi_{p,s})$ [%]
BE	0.98	0.37	0.14	1.00	0.49	4.16
FR	0.99	1.00	0.07	0.86	0.28	7.96
IT	0.34	1.00	0.12	0.52	0.26	12.58
DE	1.00	0.63	0.10	0.76	0.53	2.99
ES	0.38	0.56	0.35	1.00	0.37	10.37
UK	0.88	0.30	0.08	1.00	0.55	4.08
NO_x	industry	residential	agriculture	transport	other	$max(\phi_{p,s})$ [%]
BE	0.72	0.15	0.00	1.00	0.21	6.66
FR	0.38	0.25	0.00	1.00	0.30	7.06
IT	0.44	0.15	0.01	1.00	0.40	7.44
DE	0.80	0.18	0.00	1.00	0.20	8.93
ES	0.45	0.13	0.01	1.00	0.27	3.12
UK	1.00	0.16	0.00	0.83	0.15	7.13
SO_x	industry	residential	agriculture	transport	other	$max(\phi_{p,s})$ [%]
BE	1.00	0.13	0.00	0.00	0.01	2.29
FR	1.00	0.26	0.00	0.01	0.11	1.68
IT	1.00	0.08	0.00	0.01	0.17	2.53
DE	1.00	0.19	0.00	0.00	0.01	4.26
ES	1.00	0.18	0.01	0.00	0.14	3.87
UK	1.00	0.05	0.00	0.00	0.01	4.69
NH_3	industry	residential	agriculture	transport	other	$max(\phi_{p,s})$ [%]
BE	0.07	0.00	1.00	0.03	0.10	11.80
FR	0.02	0.00	1.00	0.03	0.03	15.03
IT	0.01	0.01	1.00	0.05	0.07	9.80
DE	0.08	0.01	1.00	0.05	0.00	20.15
ES	0.06	0.00	1.00	0.06	0.16	7.20
UK	0.04	0.00	1.00	0.13	0.15	17.48
